# IntelliSleepScorer, a software package with a graphic user interface for automated sleep stage scoring in mice based on a light gradient boosting machine algorithm

**DOI:** 10.1038/s41598-023-31288-2

**Published:** 2023-03-15

**Authors:** Lei A. Wang, Ryan Kern, Eunah Yu, Soonwook Choi, Jen Q. Pan

**Affiliations:** 1 Stanley Center for Psychiatric Research, Broad Institute of MIT and Harvard, 75 Ames Street, Cambridge, MA 02142 USA; 2 Troy High School, 2200 Dorothy Lane, Fullerton, CA 92831 USA

**Keywords:** Machine learning, Software

## Abstract

Machine learning has been applied in recent years to categorize sleep stages (NREM, REM, and wake) using electroencephalogram (EEG) recordings; however, a well-validated sleep scoring automatic pipeline in rodent research is still not publicly available. Here, we present IntelliSleepScorer, a software package with a graphic user interface to score sleep stages automatically in mice. IntelliSleepScorer uses the light gradient boosting machine (LightGBM) to score sleep stages for each epoch of recordings. We developed LightGBM models using a large cohort of data, which consisted of 5776 h of sleep EEG and electromyogram (EMG) signals across 519 unique recordings from 124 mice. The LightGBM model achieved an overall accuracy of 95.2% and a Cohen’s kappa of 0.91, which outperforms the baseline models such as the logistic regression model (accuracy = 93.3%, kappa = 0.88) and the random forest model (accuracy = 94.3%, kappa = 0.89). The overall performance of the LightGBM model as well as the performance across different sleep stages are on par with that of the human experts. Most importantly, we validated the generalizability of the LightGBM models: (1) The LightGBM model performed well on two publicly available, independent datasets (kappa >  = 0.80), which have different sampling frequency and epoch lengths; (2) The LightGBM model performed well on data recorded at a lower sampling frequency (kappa = 0.90); (3) The performance of the LightGBM model is not affected by the light/dark cycle; and (4) A modified LightGBM model performed well on data containing only one EEG and one EMG electrode (kappa >  = 0.89). Taken together, the LightGBM models offer state-of-the-art performance for automatic sleep stage scoring in mice. Last, we implemented the IntelliSleepScorer software package based on the validated model to provide an out-of-box solution to sleep researchers (available for download at https://sites.broadinstitute.org/pan-lab/resources).

## Introduction

During sleep, the body cycles through NREM (nonrapid eye movement) sleep, during which the breathing, heart rate and oscillations of the brain waves slow down, and REM (rapid eye movement) sleep, which is when vivid dreams take place. Sleep stage scoring in rodents is the process of identifying the three stages (wake, NREM, and REM) based on electroencephalogram (EEG) and electromyogram (EMG) signals. Sleep in rodents is more fragmented than that in humans and is composed of shorter episodes of NREM and REM interspaced by waking^1^. Sleep stage scoring is critical for studying sleep stage-specific measures and effects. Traditionally, sleep stage scoring has been performed manually by human experts, which is labor intensive and time consuming; thus, it is the rate-limiting step in many analyses in sleep research. To address this issue, several studies have adopted machine learning-based approaches to develop algorithms to automatically categorize sleep stages^2–6^. However, some models were developed and tested using relatively small datasets (less than 800 h of sleep recordings from 10 to 30 mice/rats)^3,5,6^, raising uncertainties regarding the generalizability of the models. Two recent studies utilized large datasets (~ 3500 h and ~ 160,000 h of sleep recordings, respectively) and developed models based on convolutional neural networks^2,4^. These models achieved high performance that was comparable to that of human experts. However, software based on these high-performing models has yet to be made publicly available for sleep researchers. In this study, we developed machine learning-based models using a large cohort of data, which consisted of 5776 h of EEG and EMG signals from 519 recordings across 124 mice. The machine learning algorithm was based on the LightGBM. The LightGBM, developed by researchers at Microsoft, is an ensemble model based on the decision tree^7^. It uses a gradient boosting approach to construct an ensemble of decision trees. The LightGBM model trained in this study consists of over 8000 decision trees. In this study, we not only demonstrated that a sleep stage scoring model based on LightGBM outperforms two widely used baseline models, logistic regression and random forest, but also showed that its performance is comparable to that of human experts. Most importantly, we confirmed that the LightGBM model is not overfitted to our data and is able to perform well on a publicly available independent set of data. To make the model freely available to academic sleep researchers without coding experience so that they can utilize it immediately, we developed a software tool, named IntelliSleepScorer, with a graphic user interface for easy access.

## Methods

This study used data collected from in vivo experiments in mice. No human experiments were involved in the study. All the experiments with animals were approved by the Institutional Animal Care and Use Committee at the Broad Institute. All experiments were performed in accordance with relevant guidelines and regulations. The ARRIVE guidelines are not applicable to this study because the focus of this study is to develop machine learning models rather than comparing different treatment groups.

### IntelliSleepScorer workflow overview

**Figure 1:**
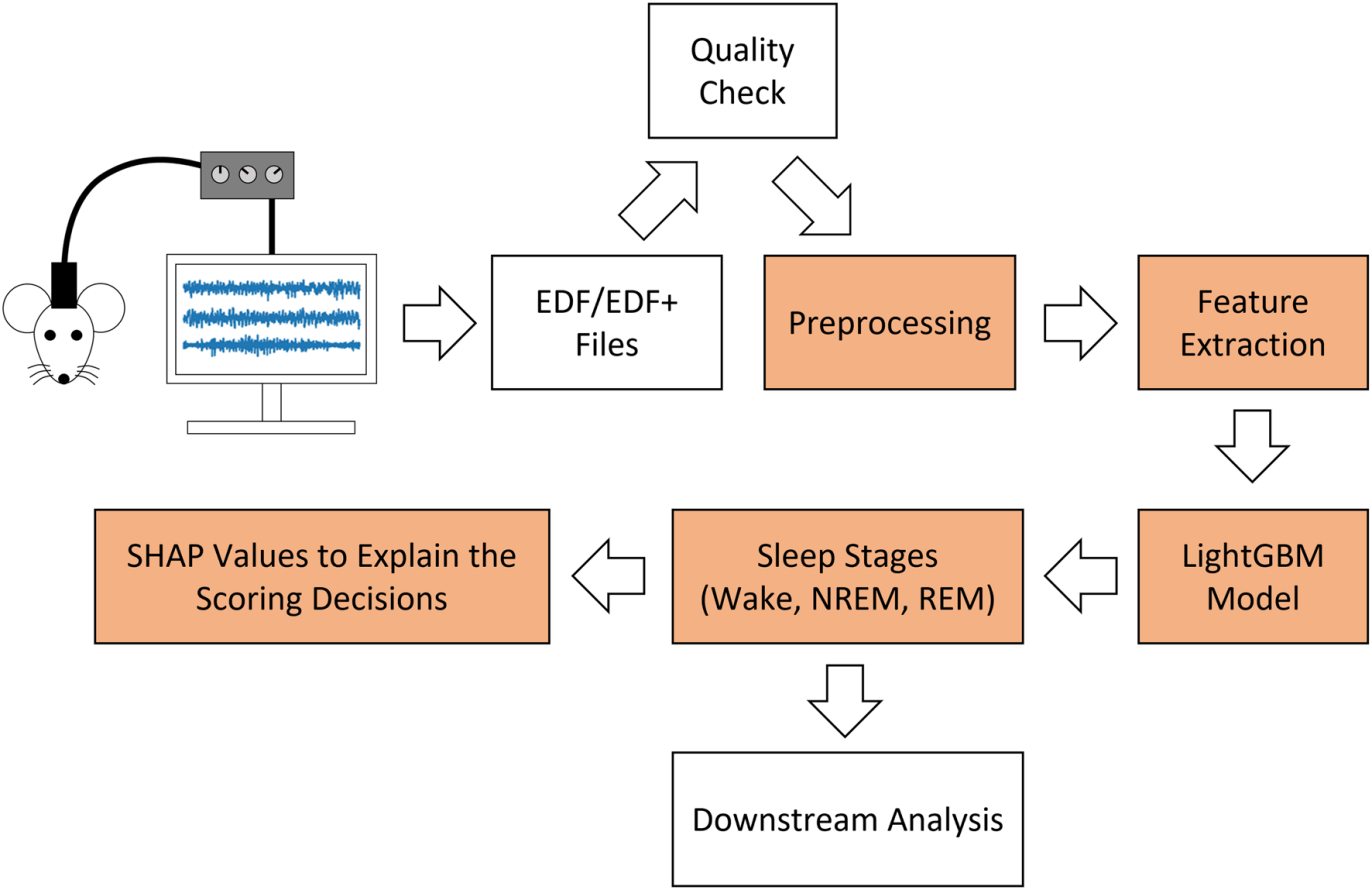
Schematic diagram of the workflow facilitated by IntelliSleepScorer. The highlighted boxes indicate the functionalities provided by IntelliSleepScorer.

The IntelliSleepScorer software is designed to enable fully automated sleep scoring in mice. Figure 1 shows the schematic view of the workflow facilitated by IntelliSleepScorer. In principle, the sleep stages generated by IntelliSleepScorer can be directly used in downstream analyses that focus on studying the physiology during Wake or NREM. If the downstream analysis requires accurately scoring REM stage, we recommend having human expert(s) verify the stages generated by the software. IntelliSleepScorer provides interactive visualizations of the signals, the associated hypnogram, and the SHAP (SHapley Additive exPlanations) values to facilitate manual verification of the generated sleep stages. SHAP values is a method based on cooperative game theory to facilitate the interpretability of machine learning models^8^. IntelliSleepScorer provides visualizations of both global SHAP values (indicating how different features values affect the scoring decision of the model in general) and epoch-level SHAP values (indicating how the features values from each epoch contribute to the scoring decision for that epoch).

### Datasets

**Figure 2:**
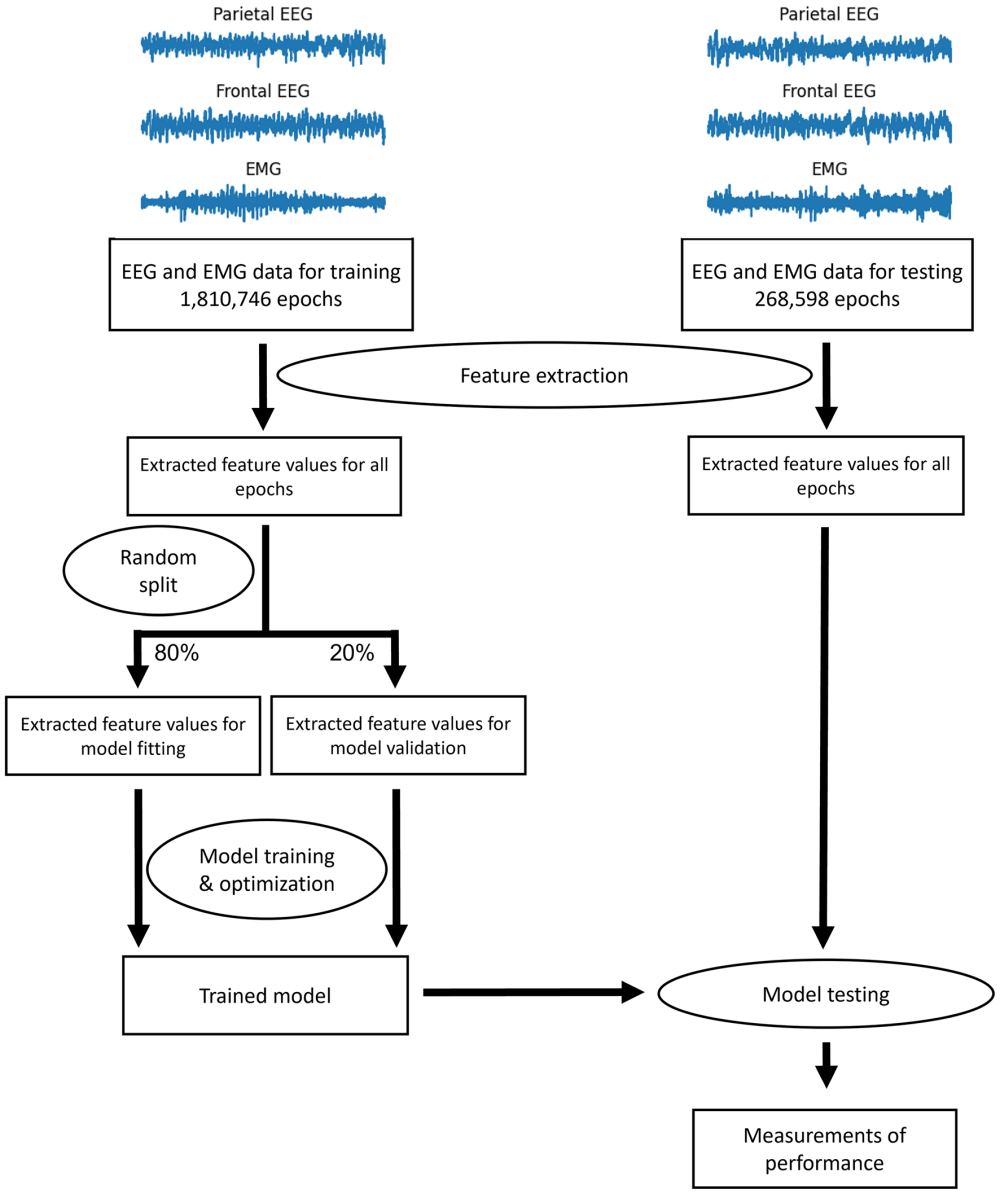
Schematic diagram of the training and testing processes for the LightGBM models.

The key properties of all datasets used in the study are listed in Supplemental Table 1. The in-house dataset consists of 519 EEG and EMG recordings (5776 h long, 301 of them were recorded during the light cycle, 218 of them were recorded during the dark cycle) from 124 C57/B6 mice with different genetic backgrounds. Each recording lasted 6–12 h. We recorded two EEG signals from the surface of the skull (EEG1 was recorded from the parietal region: AP Bregma ~ − 1.3 mm, ML Bregma ~  + 2.3 mm, and EEG2 was recorded from the frontal region: AP Bregma ~  + 1.5 mm, ML Bregma ~  + 1.5 mm) and one EMG signal placed in the nuchal muscle. The sampling frequency was 1000 Hz. The detailed method for in vivo EEG recording has been previously described^9^. The EEG and EMG recordings were divided into 10-s epochs. Models were trained and tested using epoch-level features. The processes for model training and evaluation are shown in Fig. 2. No context information (epochs before or after the target epoch) was used to train the models. The dataset contained a total of 2,079,344 epochs. The training set contained 1,810,746 epochs from 454 recordings (87.5% of all recordings, 267 of them were recorded during the light cycle, 187 of them were recorded during the dark cycle) across 111 mice. When training the LightGBM model, the training set was further randomly split into two sets; one set containing 80% of all epochs was used to fit the model, and the remaining 20% was used to validate the model. The logistic regression model and the random forest model were fitted using the entire training set. The test set contained 268,598 epochs from 65 recordings (12.5% of all recordings, 34 of them were recorded during the light cycle, 31 of them were recorded during the dark cycle) across 13 mice. The test set was never, directly or indirectly, exposed to the model during the training process. One potential route of indirect exposure was splitting multiple recordings from the same mouse into both the training set and the test set. This scenario would compromise the independence of the test dataset and lead to biased evaluations of the trained model because different recordings from the same mouse may contain individually specific patterns that could lead to overfitting. Thus, it needs to be emphasized that the data used in the test set were derived from mice that are different from those in the training set. This strategy ensures an unbiased evaluation of the performance of the trained models. The scores from human experts were used as ground truth to evaluate the performance of the models. Each epoch was associated with a ground truth label of the sleep stage that was generated by a human expert in the lab. Multiple experts were involved in the scoring process, but each recording was scored by one expert.

In addition to the in-house data, we utilized the dataset published by Miladinovic and colleagues^6^. Cohort A and cohort B from this public dataset were used to test the generalizability of the LightGBM model. The scores from these two datasets contained three categories of artifacts: wake, NREM, and REM artifacts. We merged those three categories into the wake, NREM, and REM labels to allow fair comparisons to our in-house test data since our experts did not identify artifacts during the scoring process. Cohort A includes four 24-h recordings from wild-type mice and contains 15.2% artifacts. Cohort B includes four 24-h recordings from mutant mice and contains 19.2% artifacts. Both cohorts were recorded at 128 Hz using a similar electrode setup as ours—one frontal EEG channel, one parietal EEG channel, and one EMG channel. Scores by two human experts from the same laboratories were provided for both cohorts A and B. We used cohort C, which contains eight recordings, to estimate the performance of human experts because this cohort included scores provided by two experts from different laboratories. The agreement rate among experts from different laboratories (cohort C) is a more unbiased measure of human performance compared to that among experts from the same laboratory (cohorts A and B).

### Quality control prior to feature extraction

To maximize the generalizability of the trained models, we tolerated noises and artifacts in the input data and adopted a minimal quality control process. Only two recordings were excluded from the training and validation due to loosened connections or lost signals in one of the EEG or EMG channels. Recordings were excluded when they met one of the two criteria: 1. the amplitude of either one of the EEG signals is less than 1 uV for at least 50% of the time; 2. the amplitude of the EMG signal is less than 1 pV for at least 50% of the time.

### Feature extraction

We extracted 291 features from EEG1 (138 features), EEG2 (138 features), and EMG (15 features) signals for each epoch (10 s) using the MNE-Python package and custom code. All 291 features were used to train the LightGBM model and the baseline models: logistic regression and random forest model. Only 153 features (138 EEG features and 15 EMG features) were used to train the LightGBM-1EEG model. The EEG and EMG signals were bandpass filtered at 1–40 Hz to remove 60 Hz line noise prior to feature extraction. The features extracted from the EEG signals included parameters of both the time and the frequency domains, while those extracted from the EMG signal were derived from only the time domain. There were 45 temporal features, which consisted of five raw and ten normalized attributes, for each of the three EEG/EMG channels. The raw time domain features included the mean value of the absolute amplitude, the median value of the absolute amplitude, the maximum value of the absolute amplitude, the standard deviation of the absolute amplitude, and the root mean squared value of the amplitude. The normalized features included the five raw features normalized to the per-recording mean or median values of the corresponding raw features. Normalization per-recording was performed to mitigate the inter-recording amplitude variability. Two-hundred and forty-six frequency domain features were extracted from each of the two EEG channels (123 features per channel). The frequency domain features can be divided into two sets. The first set consisted of the power from distinct frequency bands. EEG signals were transformed to the time–frequency domain using Fast Fourier Transform (FFT) algorithm implemented in the Python NumPy package. The power bands included delta (1–4 Hz), theta (4–8 Hz), alpha (8–12 Hz), sigma (12–15 Hz), beta (15–30 Hz), and low gamma (30–40 Hz) frequency bands. The ratios of power from frequency bands include the theta-delta ratio, alpha-delta ratio, sigma-delta ratio, beta-delta ratio, and low gamma-delta ratio. The second set of frequency domain features was the same as the time domain features except the data were bandpass filtered using finite impulse response (FIR) filters before feature extraction. The frequency ranges used for the FIR filters were the same as the six frequency bands described above.

### Logistic regression model

The logistic regression model used all 291 features as input to score the sleep stages. L2 penalty was used when fitting the model. The tolerance for stopping criteria was set as 1e-4. The inverse of regularization strength was set as 1. An intercept constant was added to the decision function. Lbfgs algorithm was used as the solver. The maximum number of iterations was set as 10,000.

### Random forest model

The random forest model used all 291 features as input to score the sleep stages. The number of trees was set as 100. Gini impurity was used to measure the quality of a split. The maximum depth of the tree was set as unlimited. The minimum number of samples required to split an internal node was set as 2. The minimum number of samples required to be at a leaf node was set as 1. Bootstrap samples were used when building trees.

### LightGBM model

The LightGBM model used all 291 features as input to score the sleep stages. The number of estimators was set as 100,000 and the early stopping round was set as 500. In the end, the model stopped at the 8045th round. The number of leaves was set as 100. The learning rate was set as 0.01.

### LightGBM-1EEG model

The LightGBM-1EEG model had the same overall structure and hyperparameters as the LightGBM model. The only difference was that the LightGBM-1EEG model only used 153 features (138 EEG features from one EEG channel and 15 EMG features). The LightGBM-1EEG model stopped at the 22,127th round during training.

### Metrics used to evaluate the performance of the models and human experts

The metrics used to evaluate the model performance include recall, precision, f1-score, accuracy/inter-rater agreement rate, and Cohen’s kappa score.

For each stage s ∈ {Wake, NREM, and REM}, the following formulas are used to calculate the recall, precision, and f1-score.

$${recall}_{s}=\frac{TP}{TP+FN},$$

$${precision}_{s}=\frac{TP}{TP+FP},$$

$${f1}_{s}=\frac{2}{{{recall}_{s}}^{-1}+{{precision}_{s}}^{-1}},$$

where TP indicates true positive—the number of epochs that are scored as stage s by both the model and the human expert, FP indicates false positive– the number of epochs that are scored as stage s by the model but are scored as not being stage s by the human expert, and FN indicates false negative—the number of epochs that are scored as not being stage s by the model but are scored as stage s by the human expert.

Based on the above formula, recall and precision cannot be used to compare two human experts due to the lack of ground truth. By making a random assumption (assumption 1) that one human expert is the ground truth, we can calculate the recall_assumption1_ and precision_assumption1_; if we change the assumption and pick the other human expert as the ground truth (assumption 2), we can calculate the recall_assumption2_ and precision_assumption2._ One interesting observation is that recall_assumption1_ equals precision_assumption2_ and recall_assumption2_ equals precision_assumption1._ By switching the designations of ground truth between the two human experts, we also switch the recall and precision values. Another interesting observation is that the f1 score is invariant to the switch of the designations of ground truth because it is simply the harmonic mean of the recall and precision. Therefore, we decided to use f1 scores to compare two human experts.

$$Inter-rater\,aggreement\,rate=\,\frac{Number\,of\,epochs\,agreed\,between\,two\,raters}{Total\,number\,of\,epochs}$$

Note that accuracy is equivalent to the inter-rater agreement rate between the model and the human expert.

$$Cohe{n}^{^{\prime}}s\,kappa=\frac{accuracy-{p}_{e}}{1-{p}_{e}}$$

where ${p}_{e}$ is the probability of agreement by chance. The kappa value is considered to be a better measurement of interrater reliability than accuracy because it accounts for the agreement rate by chance. Kappa values greater than 0.8 are considered to indicate nearly perfect agreement^10^.

### Software packages used in the study

All data processing, model training, and model testing were performed in the Python 3.8 environment. EEG and EMG recordings were stored in EDF/EDF + format. We used the MNE-Python package to read and process the EDF/EDF + files^11^. The scikit-learn package was used to train and test the logistic regression model and the random forest model. We used the LightGBM Python package to train and test the LightGBM model. Figures were generated using the matplotlib^12^ and seaborn packages. The Windows executable for IntelliSleepScorer as well as all the .dll files were generated using PyInstaller package.

## Results

### The LightGBM model outperformed the logistic regression and random forest models

**Figure 3:**
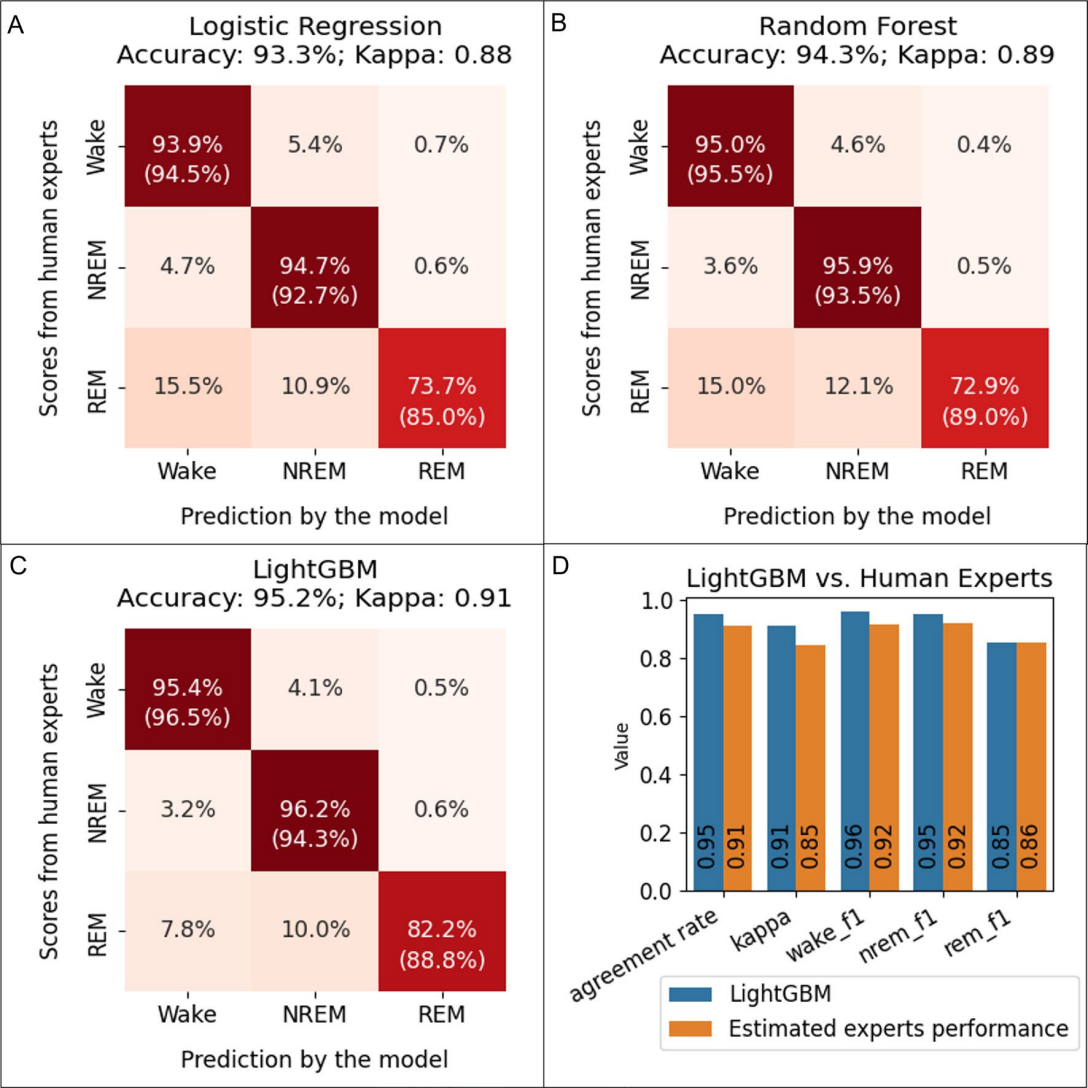
Comparisons between the baseline models (logistic regression and random forest), the LightGBM model, and human experts. (**A**–**C**): the performance of the logistic regression, the random forest model, and the LightGBM model. The numbers without parentheses in the heatmaps indicate the percentage of the scores from human experts (ground truth) that are in agreement with the scores generated by the model. The numbers inside parentheses indicate the percentage of model-generated scores that are in agreement with the scores of the human experts. Therefore, in the left–right diagonal line, the numbers without parentheses indicate recall, and the numbers inside parentheses indicate precision. (**D**) Comparisons of the accuracy, kappa, wake f1-score, NREM f1-score, and REM f1-score between the LightGBM model and human experts. The performance of human experts was estimated using the scores provided by two human experts from different labs from a publicly available dataset.

We compared the sleep stage scoring performance of the LightGBM model to two baseline models, logistic regression and random forest. Overall, all three models performed well, with accuracy greater than 93% and kappa values greater than 0.88 (Fig. 3A–C), indicating almost perfect agreement between the models and the human experts. Comparing the three approaches, LightGBM outperformed the logistic regression and the random forest models on almost all the metrics across wake, NREM, and REM stages (Fig. 3A–C). The overall accuracy was 93.3% for the logistic regression, 94.3% for the random forest, and 95.2% for the LightGBM model. The kappa was 0.88 for the logistic regression, 0.89 for the random forest, and 0.91 for the LightGBM model.

### The LightGBM model achieved human expert level performance in scoring sleep stages

We compared the performance of the LightGBM model to that of human experts. The performance of the LightGBM model was defined as the level of agreement (measured by accuracy, kappa, and f1-score) between the LightGBM model and the human experts in our laboratory. The performance of human experts was defined as the level of agreement between two experts from different laboratories in a previous study^6^. We found that the performance metrics for the LightGBM model were similar to or higher than those of the human experts (Fig. 3D). The LightGBM model had an accuracy of 0.95, a kappa of 0.91, a wake f1-score of 0.96, a NREM f1-score of 0.95, and a REM f1-score of 0.85. The two human experts from different laboratories had an agreement rate of 0.91, a kappa of 0.85, a wake f1-score of 0.92, a NREM f1-score of 0.92, and a REM f1-score of 0.86.

### The LightGBM model was highly generalizable

**Figure 4:**
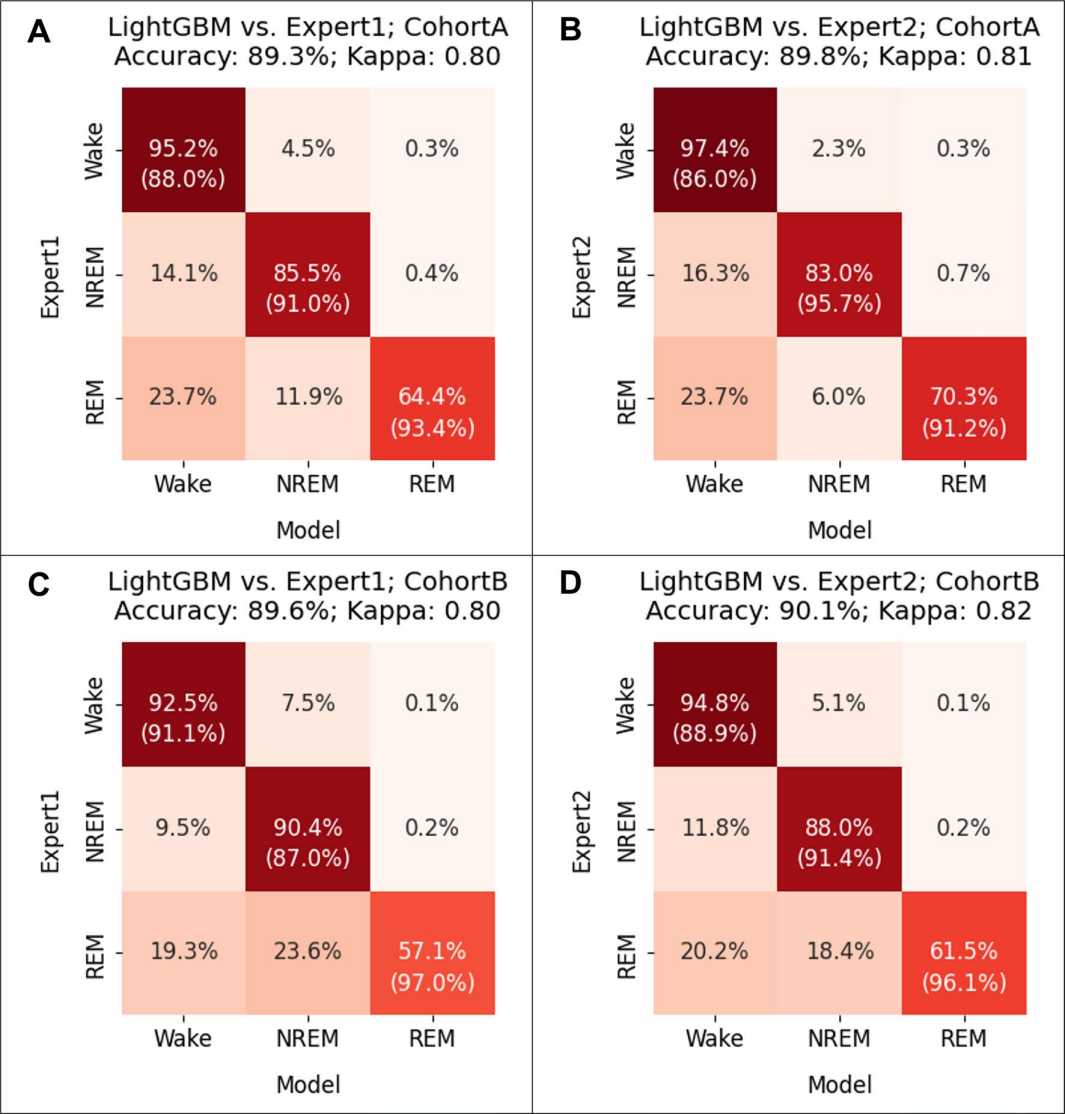
Evaluation of the generalizability of the LightGBM model using a publicly available independent dataset. The performance of the LightGBM model was evaluated across cohorts A (top row) and B (bottom row) against the scores of expert 1 (left column) and expert 2 (right column).

Using a publicly available independent dataset, we compared the sleep stages scored by the LightGBM model to the ones scored by two human experts across two cohorts of mice (four conditions). The LightGBM model achieved high performance with accuracy values close to or above 90% and kappa values greater than or equal to 0.8 in all four conditions (Fig. 4). The precision and recall values for the wake stage and the NREM stage were above 83% in all cases (16 total values) and were above 90% in more than half of the cases (9 out of 16 total values). The LightGBM model had lower recall but high precision (> 90% in all cases) when scoring the REM stage. The performance on wild-type mice (Fig. 4A, B) and mutant mice (Fig. 4C, D) was similar, suggesting that the model was able to score sleep stages for mice with different genetic backgrounds. Collectively, the high performance in an independent dataset confirmed that the LightGBM model had good generalizability.

### The LightGBM model performed consistently well on individual recordings

**Figure 5:**
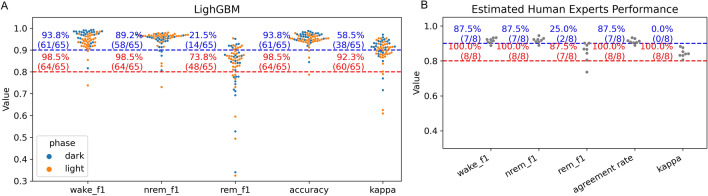
Comparisons of the recording-level performances between the LightGBM model and human experts. (**A**) The performance of the LightGBM model was evaluated individually in 65 recordings. Recordings from the dark cycle are shown in blue; recordings from the light cycle are shown in orange. (**B**) The scores of two human experts from a publicly available dataset were compared to each other across 8 recordings. Blue text indicates the percentage of recordings above the threshold of 0.9, indicated by the blue dashed lines. Red text indicates the percentage of recordings above the threshold of 0.8, indicated by the red dashed lines.

We measured the consistency of the performances of the LightGBM model across the test set of 65 recordings from 13 mice. The precision and recall values were combined into a single f1-score for easier visualization and evaluation. The results (Fig. 5A) showed high consistency for the wake f1-score (61 out of 68 or 93.8% recordings > 0.9), NREM f1-score (89.2% recordings > 0.9), accuracy (93.8% recordings > 0.9), and kappa values (92.3% recordings > 0.8). The REM f1-scores were not at consistently high levels (21.5% recordings > 0.9, 73.8% recordings > 0.8). A reduced REM f1-score was also observed when comparing the scores from two human experts (Fig. 5B). Collectively, these results demonstrate that the consistency of the LightGBM model performance reached a similar level as that of the human experts, indicating that LightGBM is a reliable tool for scoring individual recordings.

### The LightGBM model performed well across the recordings collected from light and dark cycles

The average durations of sleep stages during the light cycle are distinct from those during the dark cycle (Fig. S1), but the LightGBM model performed well regardless of the light/dark cycle (Figs. 5A, S2).

### The LightGBM model performed well on data down-sampled to 100 Hz

**Figure 6:**
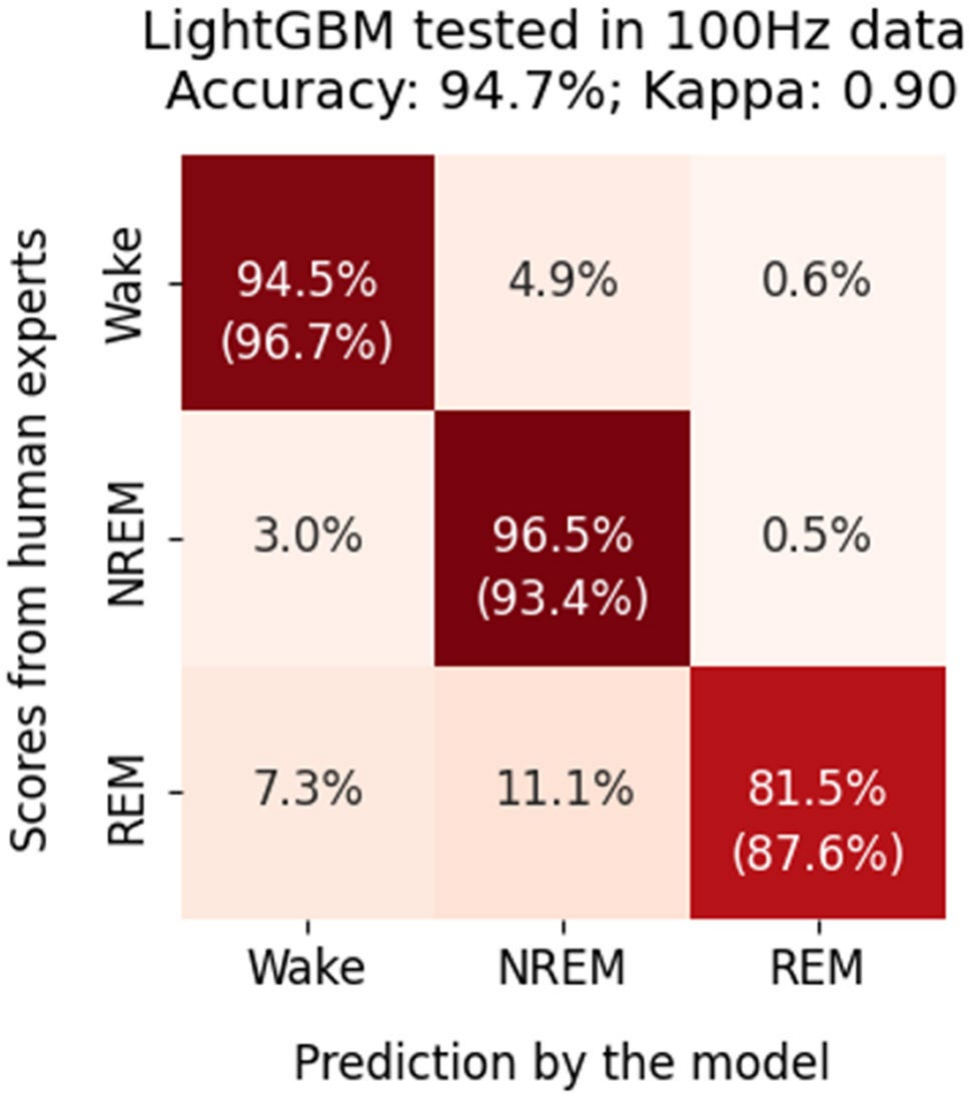
The performance of the LightGBM model when signals were downsampled to 100 Hz. The numbers without parentheses in the heatmaps indicate the percentage of the scores from human experts (ground truth) that are in agreement with the scores generated by the model. The numbers inside parentheses indicate the percentage of model-generated scores that are in agreement with the scores of the human experts. Therefore, in the left–right diagonal line, the numbers without parentheses indicate recall, and the numbers inside parentheses indicate precision.

To batch process recordings, sampling signals at a lower frequency is sometimes necessary due to limited computational resources. Here, we tested whether the sampling rate affected the performance of the LightGBM model. We down-sampled the test set to 100 Hz. The sleep stages were scored using the LightGBM model that was trained and validated using 1000 Hz data. The results showed that the performance on 100 Hz data was still high (Fig. 6). The overall accuracy was 94.7%, and the kappa was 0.90. The recall values were 94.5% for wake, 96.5% for NREM, and 81.5% for REM stages. The precision values were 96.7% for wake, 93.4% for NREM, and 87.6% for REM stages.

### The LightGBM-1EEG model performed well on data containing one EEG and one EMG channel

**Figure 7:**
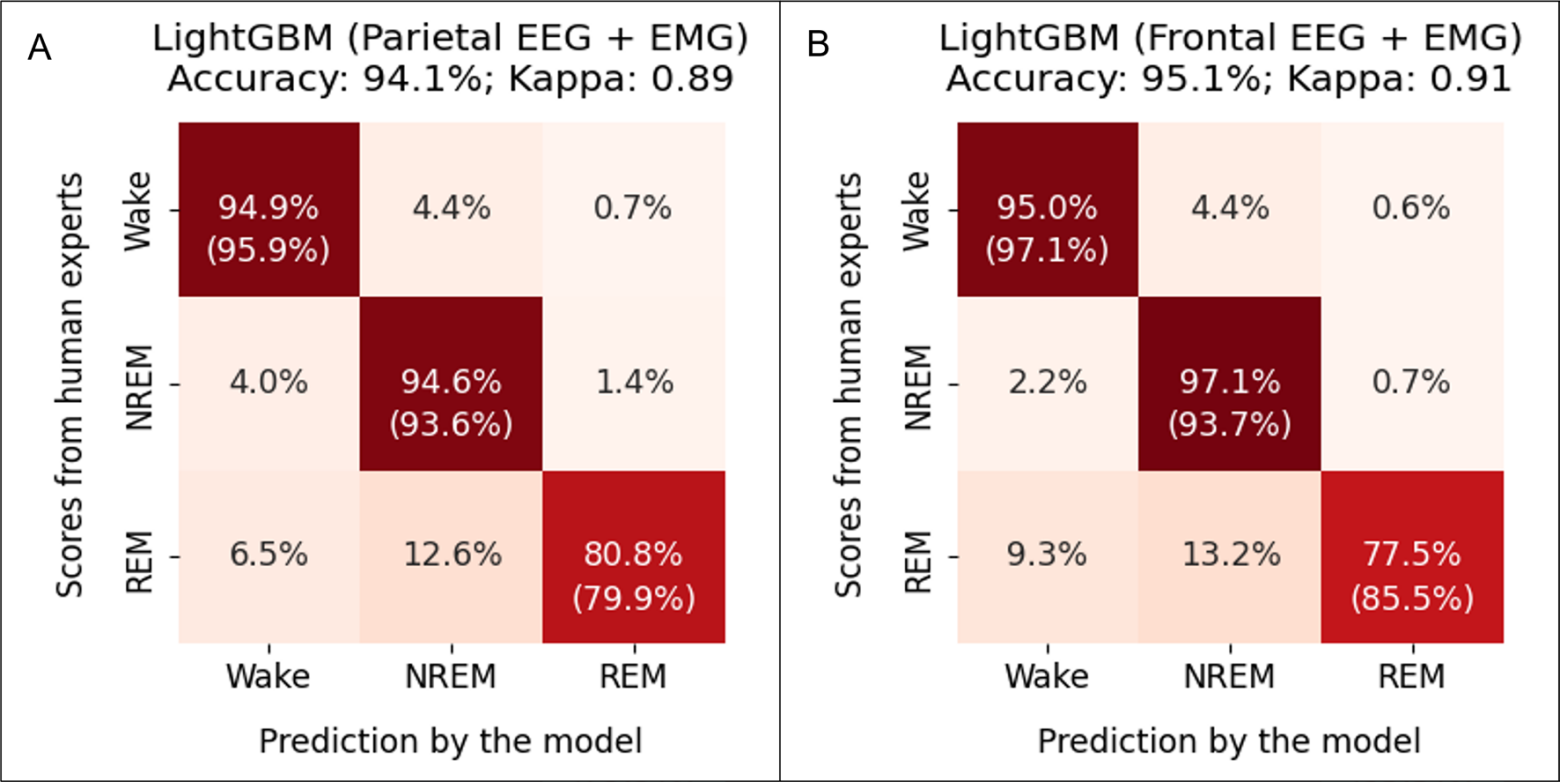
The performance of the LightGBM-1EEG model in the test data containing one EMG channel and one EEG channel in the parietal region (**A**) or in the frontal region (**B**). The numbers without parentheses in the heatmaps indicate the percentage of the scores from human experts (ground truth) that are in agreement with the scores generated by the model. The numbers inside parentheses indicate the percentage of model-generated scores that are in agreement with the scores of the human experts. Therefore, in the left–right diagonal line, the numbers without parentheses indicate recall, and the numbers inside parentheses indicate precision.

Our in vivo sleep EEG recordings included one EMG channel and two EEG channels. While this is a widely used setup, we tried to extend the utility of a LightGBM model by accommodating different recording setups. We trained a LightGBM model using only one EEG and one EMG channel (LightGBM-1EEG). The LightGBM-1EEG model still had high overall accuracy (94.1% using parietal EEG; 95.1% using frontal EEG, Fig. 7) and high kappa values (0.89 using parietal EEG; 0.91 using frontal EEG, Fig. 7). This performance was still on par with that of the human experts.

### IntelliSleepScorer implementation

**Figure 8:**
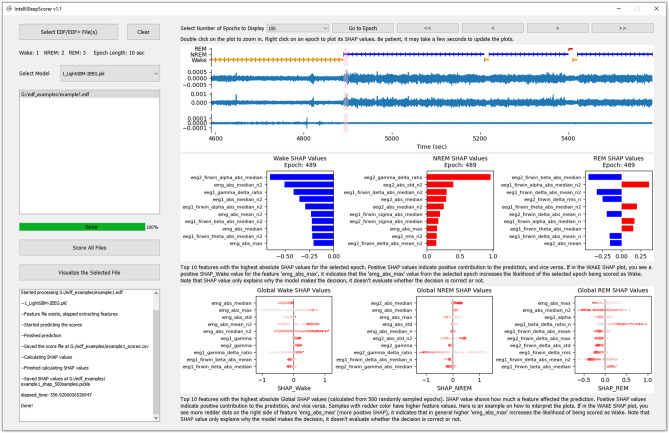
The GUI of IntelliSleepScorer. Please view the detailed documentation and instructions at https://github.com/broadinstitute/IntelliSleepScorer.

We implemented IntelliSleepScorer with a graphic user interface (GUI) to enable sleep researchers without coding experience to access and utilize the trained models. The GUI can be used to extract EEG and EMG features and score sleep stages by calling the trained models in the backend. Figure 8 shows the GUI of IntelliSleepScorer. The current version of IntelliSleepScorer accepts inputs of EDF/EDF + files. For detailed requirements of the EDF/EDF + files, please refer to https://github.com/broadinstitute/IntelliSleepScorer. The sleep stages in the output txt file are coded as 1: wake, 2: NREM, and 3: REM. On a computer (Processor: Intel(R) Core(TM) i7-8550U CPU @ 1.80 GHz 1.99 GHz; RAM: 24 GB) running on a 64-bit Windows 10 system, it took around 10 min to process 12 h of recordings sampled at 1000 Hz.

## Discussion

In this study, we demonstrated that the LightGBM model achieved an overall accuracy of 95.2% and a kappa value of 0.91, suggesting excellent agreement between the LightGBM model and human experts. Based on the study from Miladinovic and colleagues, experts from different laboratories had an agreement rate of 90% and a kappa value of 0.85 (we calculated the kappa value using the data published by the authors) in sleep stage scoring, and experts from the same laboratory had an agreement rate of 95–96%^6^. Considering that experts from the same laboratory may share the same bias, we think that the inter-laboratory agreement rate is a better indication of the performance agreement among human experts. Another study by Rytkönen and colleagues suggested that the agreement rate was 88% among three human experts and 92% between any two human experts^3^. Therefore, our LightGBM model achieved a similar level of overall performance as human experts.

Across all four models we established, including logistic regression, random forest, LightGBM, and LightGBM-1EEG, we observed a drop of performance when scoring the REM stage. For example, the LightGBM model had lower agreement with human experts in scoring the REM stage (precision, 88.8%; recall, 82.2%; f1-score, 0.85). This reduced performance when scoring the REM stage was reported for previous machine learning models^2,4^. The important question is how such performance in the REM stage compares to that of human experts. To estimate the performance of human experts in scoring the REM stage, we calculated the f1-score for the REM stage using the published sleep stage scoring results of human experts from different labs^6^. The f1-score for the REM stage was 0.86 for human experts, which is similar to the REM f1-score (0.85) of the LightGBM model. Based on these results, the overall performance of the LightGBM model for the REM stage was similar to that of human experts.

Previous studies developing deep learning models have focused on evaluating the overall performance of the trained models^2,4^. A high overall performance does not guarantee usefulness in practice if the performance of the models varies largely across different recordings. In this study, we evaluated the consistency of the performance of the LightGBM model across 65 recordings/mice individually in the test data. The results demonstrated the high consistency of the LightGBM model for scoring the wake and NREM stages during both the light cycle and the dark cycle. When scoring the REM stage, we found that 21.5% (14 out 65) of recordings had a f1-score greater than 0.9, and 73.8% (48 out of 65) of recordings had a f1-score greater than 0.8. However, the REM f1-score when comparing two human experts was not obviously higher than that of the LightGBM; 25.0% (2 out of 8) of recordings had REM f1-scores greater than 0.9, and 87.5% (7 out of 8) had REM f1-scores greater than 0.8. These results suggest that human experts also tend to have a lower agreement rate when determining REM stages. Collectively, the LightGBM model performs at a similar level of consistency as human experts across all three sleep stages. Considering that the LightGBM model performed poorly (REM f1-score < 0.6) on a few recordings, we recommend having human expert(s) verify the model-generated sleep stages if the study requires accurately scoring REM stage. In studies that focus on investigating the physiology during wake or NREM stage (such as most of our research), the LightGBM model still enables a robust and fully automated analytical pipeline.

Considering that the data generated from different laboratories may have different sampling rates, a model needs to accommodate different sampling rates to work well in practice. The performance of the LightGBM model in data sampled at 100 Hz are also on par with that in data sampled at 1000 Hz with an overall accuracy of 94.7% and a kappa of 0.90. Therefore, the performance of the LightGBM model was not affected much by the sampling frequency (as long as it is higher than 100 Hz). Using a low sampling rate has the advantages of reduced computational requirements and reduced run time for batch processing.

We demonstrated that the LightGBM-1EEG model trained using data containing only one EEG and one EMG channel had high performance with an overall accuracy greater than 94.1% and kappa greater than 0.89. The ability to perform well using only one EEG instead of two further expanded the generalizability of our scoring tool since any data containing at least one EEG and one EMG channel can be reconstructed to meet the requirement of the LightGBM-1EEG model.

In conclusion, we developed the LightGBM model, which achieved a similar level of performance in sleep stage scoring compared to human experts. We validated the generalizability of the LightGBM models extensively for practical use in research. In addition, we implemented IntelliSleepScorer, a GUI software based on the models reported in this study. Such automation in sleep scoring has an immediate impact for sleep researchers who would like to quickly score large amounts of EDF/EDF + files without in-depth coding experience.

## Supplementary Information

Supplementary Information 1.

Supplementary Information 2.

Supplementary Information 3.

## Data Availability

IntelliSleepScorer software was released under the Creative Commons Attribution-NonCommercial-ShareAlike (CC-BY-NC-SA) license. It is free to academic users at this link (https://sites.broadinstitute.org/pan-lab/resources). For commercial use, please contact the authors for licenses. The source code is available at https://github.com/broadinstitute/IntelliSleepScorer.
