# Supplementary material for: IntelliSleepScorer, a software package with a graphic user interface for automated sleep stage scoring in mice based on a light gradient boosting machine algorithm
Source: Sci Rep. 2023 Mar 15;13:4275. doi: 10.1038/s41598-023-31288-2 (PMC10017698; doi:10.1038/s41598-023-31288-2)
Supplement: Supplementary file 1 — Supplementary Information 1. [file 41598_2023_31288_MOESM1_ESM.pdf]

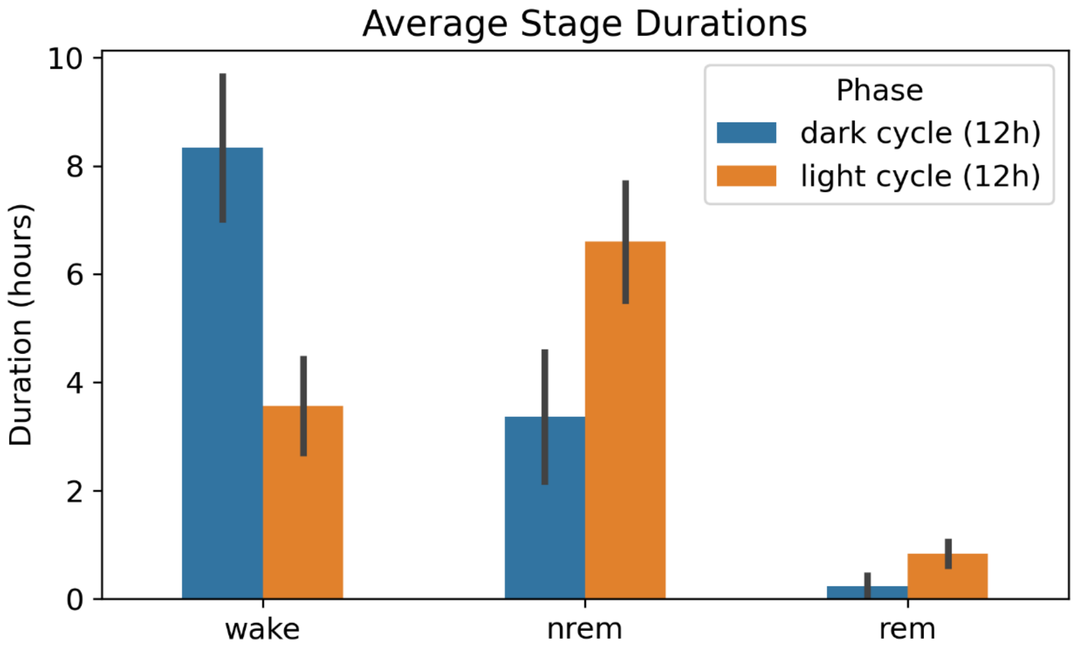


Figure S1. The average duration of different sleep stages during light and dark cycles. Error bars indicate standard deviation.

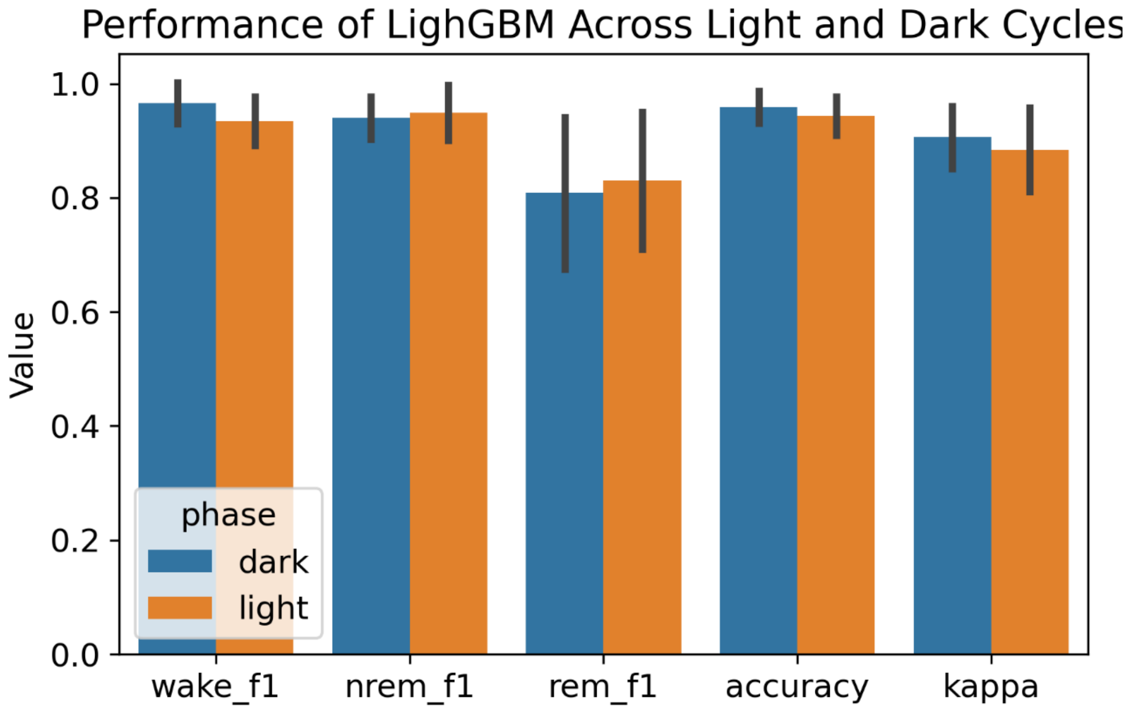


Figure S2. The LightGBM model performed well across the recordings collected from light and dark cycles. Error bars indicate standard deviation.

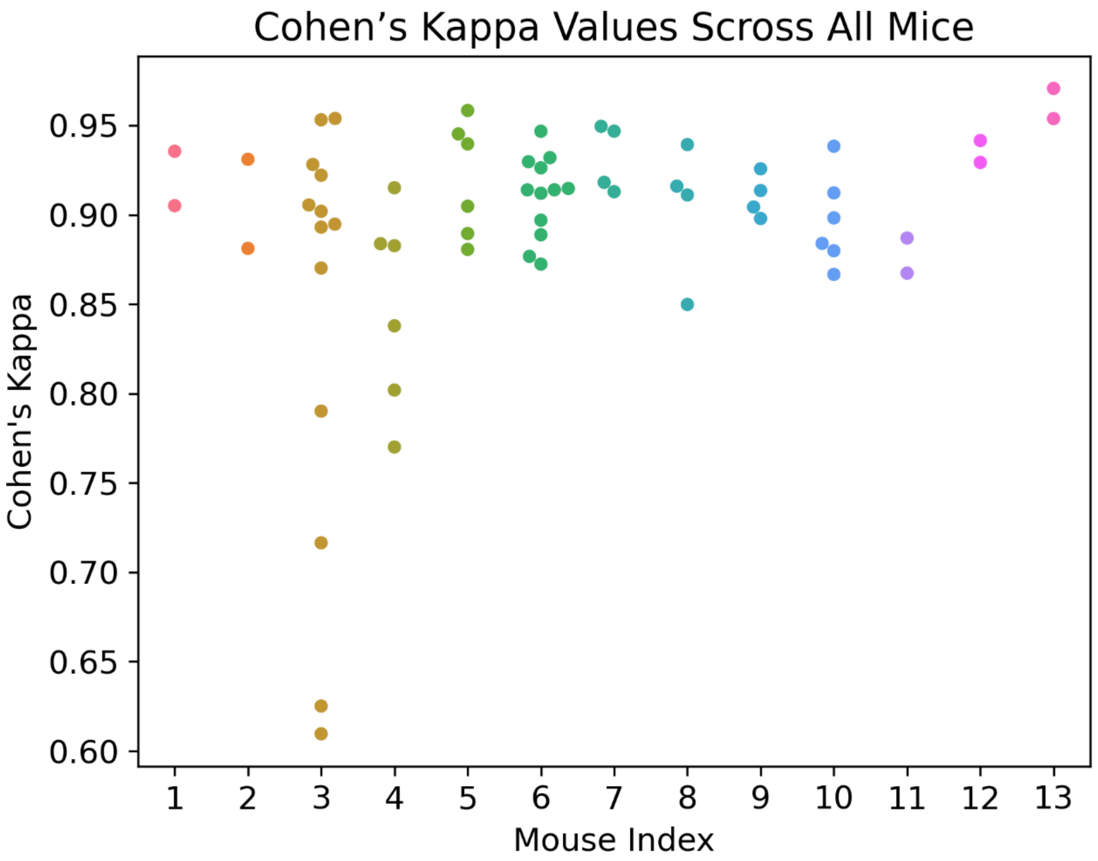


Figure S3. Cohen’s Kappa values across 13 mice and 65 recordings from the test data.

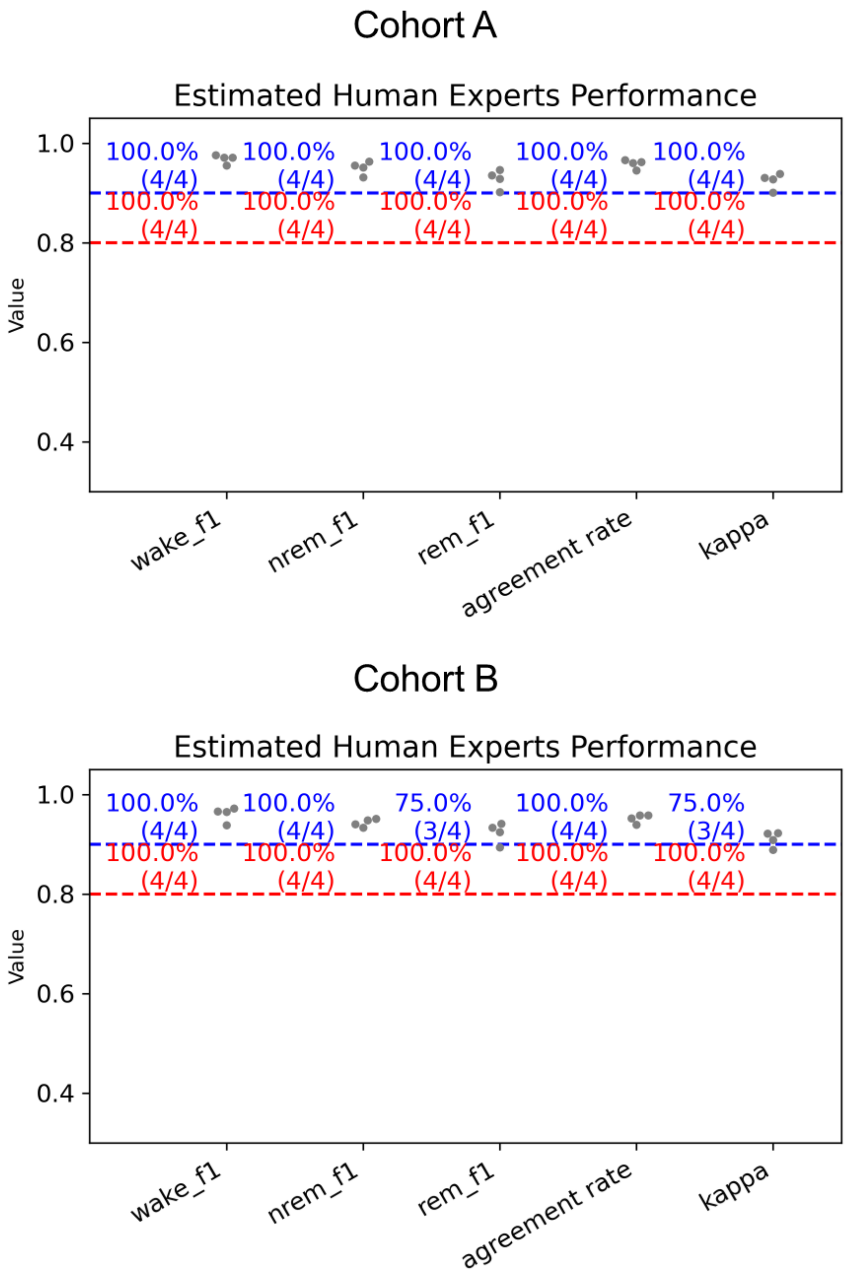


Figure S4. The estimated performance of human experts using cohort A and cohort B from the previous study by Miladinović et al[1].

## References


1. Miladinović Đ, Muheim C, Bauer S, et al. SPINDLE: End-to-end learning from EEG/EMG to extrapolate animal sleep scoring across experimental settings, labs and species. *PLOS Comput Biol*. 2019;15(4):e1006968. doi:10.1371/journal.pcbi.1006968
